# The effect of size and sex ratio experiences on reproductive competition in *Nicrophorus vespilloides* burying beetles in the wild

**DOI:** 10.1111/jeb.12803

**Published:** 2016-01-08

**Authors:** P. E. Hopwood, A. J. Moore, T. Tregenza, N. J. Royle

**Affiliations:** ^1^ Centre for Ecology and Conservation, Biosciences, College of Life & Environmental SciencesUniversity of ExeterCornwall CampusPenrynUK; ^2^ Department of GeneticsUniversity of GeorgiaAthensGAUSA

**Keywords:** body size, competitive investment, parental investment, sex ratio, sexual selection

## Abstract

Male parents face a choice: should they invest more in caring for offspring or in attempting to mate with other females? The most profitable course depends on the intensity of competition for mates, which is likely to vary with the population sex ratio. However, the balance of pay‐offs may vary among individual males depending on their competitive prowess or attractiveness. We tested the prediction that sex ratio and size of the resource holding male provide cues regarding the level of mating competition prior to breeding and therefore influence the duration of a male's biparental caring in association with a female. Male burying beetles, *Nicrophorus vespilloides* were reared, post‐eclosion, in groups that differed in sex ratio. Experimental males were subsequently translocated to the wild, provided with a breeding resource (carcass) and filmed. We found no evidence that sex ratio cues prior to breeding affected future parental care behaviour but males that experienced male‐biased sex ratios took longer to attract wild mating partners. Smaller males attracted a higher proportion of females than did larger males, securing significantly more monogamous breeding associations as a result. Smaller males thus avoided competitive male–male encounters more often than larger males. This has potential benefits for their female partners who avoid both intrasexual competition and direct costs of higher mating frequency associated with competing males.

## Introduction

In species that provide parental care, females often provide the bulk of care while males tend to spend more time seeking out further mating opportunities (Kokko & Jennions, 2012). One factor that may influence the pattern of these sex role differences is the ratio of females to males in the population. Most sexual species produce females and males in similar numbers (primary sex ratio), but biases in the adult sex ratio (ASR) due to sex‐specific mortality and/or reproductive ‘time‐out’ differences can affect the ratio of available, receptive mating partners: the operational sex ratio (OSR) (Emlen & Oring, 1977; Kvarnemo & Ahnesjö, 2002). Population sex ratio bias, by affecting how difficult it is to find a mate, can influence the intensity of mating competition (Clutton‐Brock & Vincent, 1991; Andersson, 1994). This alters the balance between the cost and benefit of caring for offspring, vs. trying to secure more mating partners (Fromhage *et al*., 2007; Kokko & Jennions, 2012).

Theoretical models incorporating the OSR generate contrasting predictions for sex‐specific patterns of parental investment that may depend on ecological context. One prediction is that with an excess of males in the population, the strength of sexual selection for competitive ability should increase due to intensified male–male competition over mating (Trivers, 1972; Emlen & Oring, 1977; Kvarnemo & Ahnesjo, 1996). However, intensified mating competition need not lead to increased investment in competitive traits because avenues other than fighting for mates are available to males, including increasing parental investment (Kokko & Jennions, 2012). Recent work highlights the importance of considering other factors whose inclusion in models may alter predictions (e.g. Kokko & Jennions, 2008; Alonzo, 2010; Kokko *et al*., 2012). Males may gain more on average by maximizing returns from a realized breeding opportunity (i.e. by being good parents) than by searching for potentially contested future mating opportunities. The value of investing in mating vs. parental care depends on the slope of relationship between an individual's fitness and number of additional matings (Kokko *et al*., 2012) and on the relationship between parental care and offspring survival/fitness and paternity (Kvarnemo, 2006; Kahn *et al*., 2013b).

Sexual selection favouring male traits that improve competitive success could result in a subset of males that have more to gain than others by deserting parental duties to capitalize on their enhanced potential mating success (Kokko & Jennions, 2008). The corresponding response of less favoured males might be to invest more heavily in parental care whenever they get the opportunity. However, ecological context can shift parental investment decisions from model predictions (Kvarnemo & Ahnesjo, 1996). For example, breeding resources other than mates may be the limiting factor for reproductive success. Scarce nesting sites can lead to sex role reversal in some fish, (e.g. blennies, *Salaria pavo*) leading to females courting males instead of males courting females and intrasexual aggression in both sexes (Almada *et al*., 1995). In addition to scarcity, breeding resource unpredictability may also alter the costs and benefits of parental decisions or select for plasticity where there is no single optimal strategy (Shine & Brown, 2008). Sex ratio itself can be a limiting factor for reproductive success. For example, Kahn *et al*. (2013a) showed that a proportion of autumn‐born female mosquitofish, *Gambusia holbrooki*, overwinter and breed again alongside their offspring in spring while males perish after each breeding season. This intersexual differential mortality results in a cyclical sex ratio bias that has led to ‘anticipatory’ biased sex allocation (Kahn *et al*., 2013a).

There has been little theoretical modelling of plasticity in paternal care investment in response to short‐term changes in intensity of reproductive competition. Adaptive plasticity might be favoured if cues (e.g. patterns of seasonal variation in competitive intensity or encounter rates) reliably predict the relative reproductive value of pursuing mating opportunities at the expense of time spent caring for offspring within the timescale of an individual's lifespan. In this case, early social encounters predicting likely future mating opportunities and/or the strength of competition might affect lifetime patterns of male behaviour (Kasumovic & Brooks, 2011). Reproductive pay‐offs are also likely to be mediated by the likelihood of success in mating competition (e.g. through being relatively large or small among competitors) (Kokko & Jennions, 2008).

In a previous study, we showed that male burying beetles, *N*.* vespilloides*, alter the amount of time they stay and provide parental care in response to variation in direct reproductive competition over breeding resources (Hopwood *et al*., 2015). In this context, males altered biparental investment in ways that appear to provide potential current and future paternity benefits (Hopwood *et al*., 2015). However, in addition to contesting breeding carcasses, burying beetles may encounter one another when they feed together on carrion or when they are attracted to males releasing pheromones to attract mates (when they may not have a carcass themselves) (Eggert & Müller, 1997; Scott, 1998; Müller *et al*., 2007). When they meet, burying beetles detect cuticular chemicals expressed by one another that transfer information about sex and status (Steiger *et al*., 2009). Thus, social encounters might provide information about relative competitive ability or likely intensity of competition sufficient for individuals to use as cues to optimize future breeding behaviour.

In this study, we test the hypotheses that sex ratio cues prior to breeding, and male body size (a trait influencing competitive ability) would affect future parental care behaviour, either independently or in tandem, in male burying beetles, *N. vespilloides*, in a natural setting. To test our hypotheses, we manipulated sex ratio cues during early life (i.e. prebreeding) in male beetles and then provided them with a carcass in the field, which necessitated them calling breeding partners and facing potential competition from wild beetles for the breeding resource. Recent studies provide evidence to support the idea that male attendance in biparental care in *N. vespilloides* is largely self‐serving, leading to strong sexual conflict, perhaps influenced by certainty of paternity or other male benefits and not by improved offspring fitness (Benowitz *et al*., 2013; Hopwood *et al*., 2015; Parker *et al*., 2015). Males reared with cues indicating high intensity of male competition (i.e. male‐biased sex ratio) could benefit from extending the duration of parental care because mating opportunities elsewhere are likely to be reduced. This is because males repeatedly mate with females that are attracted to the carcass, providing a potential paternity benefit in both the current breeding bout and in future broods that involve these mated females (Hopwood *et al*., 2015).

We therefore predicted that males reared with cues indicating a high intensity of male competition should prolong parental care compared to those males that experienced a female‐biased sex ratio during early adult life. We also predicted that larger males would desert broods before smaller males. In burying beetles larger, males have a strong competitive advantage over smaller males and are more likely to achieve dominant status in contests over vital breeding carcasses (Otronen, 1988; Hopwood *et al*., 2013; Lee *et al*., 2013). As a result, males of different sizes may behave differently given the same information. Because larger males have a greater likelihood of dominating future contested reproductive resources, they might be able to exploit potential benefits of earlier desertion from parental duties. Any benefits of deserting early may be harder to achieve for smaller males because they are more sensitive to variation in the social competitive environment as they are more likely to lose in competitive encounters with rival males (see Hopwood *et al*., 2014).

## Materials and methods

### The study species

In burying beetles, the relationship between mating success and reproductive success is defined by the availability of carcasses because a small vertebrate carcass is necessary to rear each brood of offspring (Eggert & Müller, 1997). This in turn is mediated by variation in individual success (within both sexes) in locating and competing for suitable carcasses (Eggert & Müller, 1997). For male burying beetles, the relationship between mating success and reproductive success differs between two mating contexts that correspond to alternative mating strategies (Müller *et al*., 2007). Firstly, males may call (by emitting pheromones), and mate with females despite the absence of a carcass. In this context, the number of mating partners is likely to be important because a proportion of these mated females may subsequently locate a carcass and reproduce using stored sperm. Secondly, a male may benefit from increased paternity by being the dominant (or only) male on a carcass (House *et al*., 2007; Müller *et al*., 2007). In this second context, he has the opportunity to maximize the precedence of his sperm against that of competitors (present or absent) by repeatedly mating with the female(s) while keeping any subordinates away (Müller & Eggert, 1989; Hopwood *et al*., 2015). Because both sexes search for breeding carcasses, the sex ratio of individuals contesting a single carcass can be biased in either direction and the intensity of competition during a single breeding attempt depends on the number of beetles attracted to each carcass. Both sexes can provide effective post‐natal care, but burying beetles maintain a female‐biased care pattern (Eggert & Müller, 1997). A small majority of breeding events appear to be biparental, although males usually desert before females and sometimes even before postnatal care commences (Eggert & Müller, 1997). The next most common breeding association is female uniparental care (prenatal and post‐natal care) while male uniparental post‐natal care is the least common (Eggert & Müller, 1989; Eggert, 1992; Müller *et al*., 2007).

### Experiment 1

To minimize potential confounding effects both of unintentional laboratory selection and experiential variation among individuals, we generated F1 stock for use in this experiment. A total of 669 wild beetles were caught in funnel‐type bottle traps baited with rotten salmon on a weekly basis throughout the spring and summer of 2012. We removed phoretic mites (by blowing with air) and fed captured beetles on decapitated mealworms *ab libitum* for 1 week. Within the pool of beetles captured each week, pairs were randomly assigned a mouse carcass on which to breed.

### Social cue manipulation

In total, 307 of the resulting F1 progeny were randomized within blocks, corresponding to the weeks their parents were trapped, and assigned to one of four treatment groups producing 91 experimental males that had experienced a manipulated sex ratio cue (i.e. one male from each group). The four sex ratio cue treatments consisted of (i) female bias: three females and one male (mfff); (ii) male bias: one female and three males (mmmf); (iii) no sex‐bias: two females and two males (mmff); (iv) absence of cue: a single virgin male (m). These groups of beetles were housed together for 2 weeks from eclosion in translucent plastic boxes 32 × 18 × 12 cm with 3‐cm moist compost and egg cartons to provide three‐dimensional structure allowing places to hide and calling platforms. Boxes were kept on shelves outside the laboratory to minimize differences in seasonal photoperiod and climactic experience between experimental males and the wild beetles with which they would interact (the field site is 2 km from the laboratory). Experimental groups were fed decapitated mealworms at the rate of two per individual twice weekly. These conditions provide a benign environment where individuals can encounter one another but do not have to compete for limited resources while undergoing sexual development. All individuals were initially weighed, measured and marked (using Indian ink dots on elytra, see Hopwood *et al*., 2015), and after 2 weeks, a single male was chosen (randomized in relation to markings in treatments with more than one male), removed from each container and used as the experimental beetle.

### Field methods and materials

400 mm lengths of black PVC‐U, 110 mm Ø pipes (‘Nicrocosms’) were buried vertically up to 100 mm with three 40 mm Ø exit and entry ports surrounding the structure positioned at ground level (See Hopwood *et al*., 2013 for details). These ports were extended with 40 mm Ø PVC‐U pipe to protrude 100 mm and reduce the ingress of light to the interior space. Six further ventilation holes (10 mm Ø) were drilled towards the top of each Nicrocosm. Infrared surveillance cameras (N08CX night vision CCTV camera; Maplin, Rotherham, UK) using motion detecting software (AVerMedia NV6240 Express, DVR version 7.7.0.0007; www.avermedia-dvrs.com) were positioned within to capture beetle activity around a mouse carcass that was positioned inside and a small inverted red polypropylene bucket protected the interior space and from rain and light.

Starting from early July to mid September 2012, we placed a single experimental male in each Nicrocosm with six or fewer replicates running concurrently, spaced at least 20 m apart, in our study area of mixed deciduous woodland (approximately 0.5 ha: coord: N50°11′42″, W5°07′51″). Each Nicrocosm contained a 24‐h thawed mouse carcass that the beetle(s) inside were free to bury completely. Allowing beetles to conceal mice within the filmed arena still permits observations of above ground activities such as fights and outcomes, desertion and arrivals. Single, marked experimental males were introduced to Nicrocosms at approximately midday whereupon they invariably took cover in the soil substrate before emerging (usually about 16:00) to explore later in the day during the species’ normal activity period. Each replicate ran until at least 6 h after the desertion of the experimental male was witnessed (assessed as the male observed leaving the Nicrocosm by a port and not returning). Males and females that had been engaged in parental care often unfurled their wings prior to leaving voluntarily, perhaps in readiness for flight; this behaviour was not witnessed in beetles that were evicted forcibly by others. Of a total of 91 experimental males placed in Nicrocosms in experiment 1, 76 were witnessed calling before the arrival of wild nonexperimental beetle(s). We found unutilized carcasses attracted and were devoured by large slugs (predominantly *Arion* spp.) and often become unusable for breeding by burying beetles after two or three nights as a result (in 11/91 instances, no beetle(s) arrived after two successive evenings of males calling and these carcasses were lost to slugs). A total of 3/91 replicates failed because the experimental male deserted without establishing contact with the carcass and one male called but the times of subsequent arrivals were not clear from footage obtained. Of the 76 calling males that attracted wild beetles, 13 failed to attract beetles until the second night of calling. To minimize uncertainty in our attribution of the stimulus to which arrivals responded (e.g. volatile products of carcass decomposition vs. male pheromone emission), these 16 males were excluded from analyses of patterns of wild beetle arrivals. This left 63 males that successfully attracted beetles after calling on their first day, and these were used in all the analyses of arrival times and sex of arriving beetles. No additional burying beetles were witnessed arriving after carcass burial was complete during this experiment and none of the four congeneric species was seen in the Nicrocosms (easy to distinguish visually because in the UK only *N. vespilloides* has black, rather than red, antennae tips).

### Behavioural observations

We defined first contact with the carcass as the time of first exploration of the carcass surface by a male, rather than physical contact incidental to hiding or running past. Male calling was assumed when males ceased walking and adopted a characteristic and unambiguous *sterzeln* posture: tail‐up, head‐down with abdominal segments extended (Pukowski, 1933; Müller & Eggert, 1987). We measured time until experimental males deserted as total pre‐ and post‐natal attendance, defined as time between arrival of the first wild nonexperimental female and the time when the experimental male left. Only experimental males that were witnessed participating in a biparental partnership, by virtue of them being either the only male, or successful in repelling rivals, were included in the analysis of time until desertion. This is because usurped and defeated rival males, although often adopting a satellite role and sneaking matings, do not remain to provide care for the brood. We exhumed carcasses after experimental male desertion to confirm his absence, to corroborate the number and sex of any remaining beetles and to ensure that the reproductive bout was successful (i.e. larvae were produced).

### Experiment 2

To determine the proportion of males and females that initially locate and compete for carcasses, we placed 194 mouse carcasses in Nicrocosms at the field site in the summer of 2013. There is evidence that the size of carcasses may affect male calling behaviour in some burying beetles (e.g. Trumbo & Eggert, 1994), so all carcasses across both experiments were standardized and weighed between 19.9 and 21.9 g. We used the spatial placement protocol as described above but reviewed video footage each morning to determine the carcasses that had attracted beetles. Utilized carcasses were disinterred the day after burial and the beetles sexed, weighed and measured and categorized by the order that they arrived by matching beetle size, markings and mating and/or fighting behaviour witnessed through video footage. Carcasses remaining unclaimed and unburied were discarded on the third day and replacements sited in different locations. We also sexed, measured and weighed a sample (*n *= 1952) of the local population of burying beetles throughout 2013 (2nd May to 17th September) by setting 12 baited traps each week in woodland approximately 1 km from the study site. These beetles were released back at the capture site in the same week as they were retrieved (we have unpublished evidence that recapture rate is approximately 17% overall in these circumstances and is not related to body size).

### Statistics

All analyses were performed using ‘R’ version 3.1.3 (R Development Core Team 2011). The time lag between male commencing calling time and the first beetle arriving (square root transformed) and male time until desertion (i.e. the duration of his biparental association with his partner) were analysed as response variables with linear models including treatment and male body size (pronotal width) and their interaction as explanatory variables. The sex of the first arriving beetle, whether or not males secured a monogamous breeding association (yes or no) and whether or not male–male competition or female–female competition occurred at a carcass (yes or no) were analysed as response variables with generalized linear models using a binomial error structure and social cue treatment and calling male body size as explanatory variables.

For experiment 2, the sex ratio of first arrivals was tested against an expected 0.5 using a χ^2^ goodness‐of‐fit test. We categorized beetles by sex and according to whether they were first arrivals or subsequent arrivals (i.e. either to a male already arrived and calling or to a carcass without a male present). Mean pronotum width in millimetres among these four groups was analysed using anova with *post hoc* Tukey's honest significant difference. Unless stated, means are presented ± 1 standard error throughout.

## Results

### Experiment 1

There was no statistically significant difference in size of males placed on carcasses in the field among the four treatments [linear model (LM) pronotum, *F*_3,87_ **= 0.971 *P *= 0.410; grand mean ±SD* *= 4.84 ± 0.35 mm]. A total of 88% of all carcasses (*n *= 80/91) were discovered in the first 2 days; 95% of these wild nonexperimental beetle arrivals apparently responding to calling experimental males (*n *= 76/80). Of the 63 males that attracted beetles on the first night of calling, the average time from start of the experimental male calling until the first wild nonexperimental beetle arrived was 79 ± 59 min (mean ± SD). Time until desertion for experimental males that were not usurped and thus became caregivers was 249 ± 52 h (mean ± SD), and there was no statistically significant difference among social cue treatments (LM, *F*_3,50_ **= 1.823, *P *= 0.155), or in relation to calling male size (*F*_1,49_ **= 0.101, *P *= 0.752). The interaction between social cue treatment and calling male size was also not significant (*F*_3,46_ **= 0.078, *P *= 0.972).

**Figure 1:**
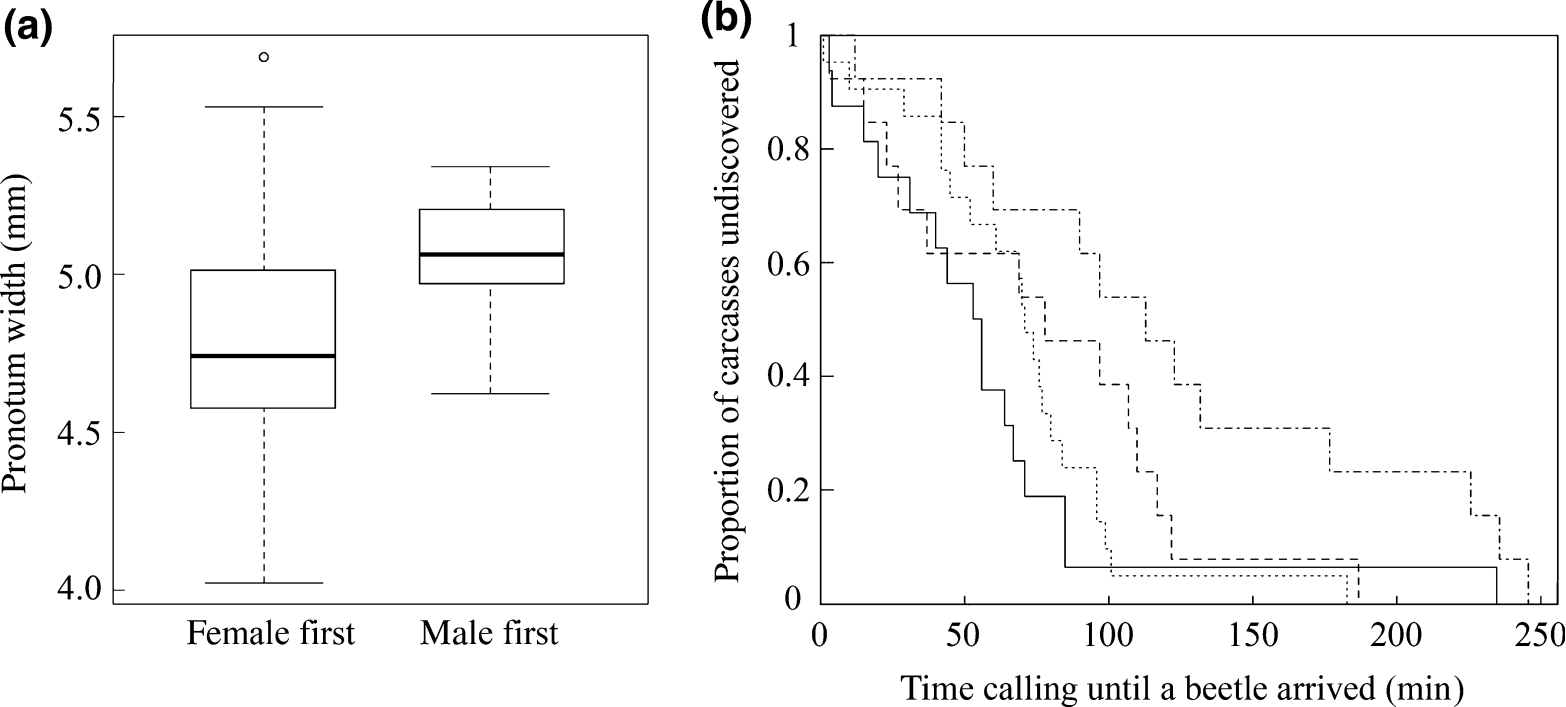
(a) Box plot (to illustrate actual distribution using medians and IQRs; see Weissgerber *et al*., 2015) showing the relationship between calling experimental male size (*y*‐axis) and whether males first attracted a female or a male (*x‐*axis); (b) ‘survival’ curve to illustrate the difference in time elapsed (*y‐*axis), between experimental male commencing calling and first beetle arrival, among treatment groups (‘m’= solid line; ‘mmff’= dotted line; ‘mfff’= dashed line; ‘mmmf’= dot‐dash line).

A higher proportion of females than males overall arrived first to calling males [(48 female beetles vs. 15 males), binomial test, *P* < 0.0001]. The sex of the first arrival was associated with the size of the calling male: smaller males were more likely to attract a female first than were larger calling males [generalized linear model (GLM), binomial, *χ* ^2^_58_ **= 7.354, *P *= 0.007, Fig. 1a]. There was no statistically significant effect of social cue treatment on the sex of first arrivals (*χ* ^2^_58_ **= 2.592, *P *= 0.459) and no statistically significant interaction between calling male size and social cue treatment (*χ* ^2^_55_ **= 5.905, *P *= 0.116). However, males in social cue treatment ‘mmmf’ (i.e. from a rearing environment with a sex ratio of three males and one female) took longer on average to attract a beetle of either sex than did males in other social groups (LM, *F*_3,58_ **= 2.851 *P *= 0.045, Fig. 1b). The time taken for a wild beetle of either sex to arrive was not related to the size of the calling male (*F*_3,58_ **= 0.001 *P *= 0.978), and nor was there any effect of the interaction between calling male size and social cue treatment (*F*_3,55_ **= 0.197 *P *= 0.898).

**Figure 2:**
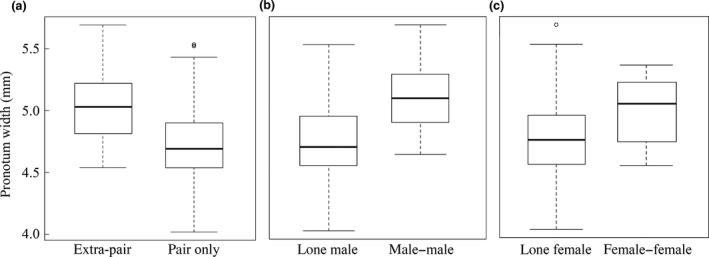
Box plots (to illustrate actual distribution using medians and IQRs) showing the relationship between experimental male size (*y‐*axes) and the competitive environment they encountered after calling for a mate (*x‐*axes): (a) size distribution of males encountering extra‐pair competition (male and/or female) vs. encountering a single female only; (b) ‘lone male’: experimental male was the only male (regardless of female number), ‘male–male’: experimental male meets at least one male rival; (c) ‘lone female’: females encounter no female rivals, ‘female–female’: female meets at least one female rival.

There was a significant association between the size of calling experimental males and the probability of subsequent competition for his carcass: males achieving uncontested monogamous pairings were smaller than those whose ownership of carcasses was challenged by the arrival of extra‐pair competitors (GLM, binomial, *χ* ^2^_56_ **= 12.687, *P *= 0.0004, Fig. 2a). As a result, small males encountered fewer male rivals and experienced less male–male competition than did large males (GLM, binomial, *χ* ^2^_56_ **= 14.003, *P *= 0.0002, Fig. 2b). There was a similar relationship between the experimental male's size and the probability of female–female competition: carcasses where there was more than one female competing were associated with larger males on average than those where the first arriving female had no female rivals (GLM, binomial, *χ* ^2^_56_ **= 3.909, *P *= 0.048, Fig. 2c). There were no statistically significant effects of social cue treatment or the interaction between social treatment and calling male size in any of the three latter analyses described above (all *P *> 0.189).

### Experiment 2

**Figure 3:**
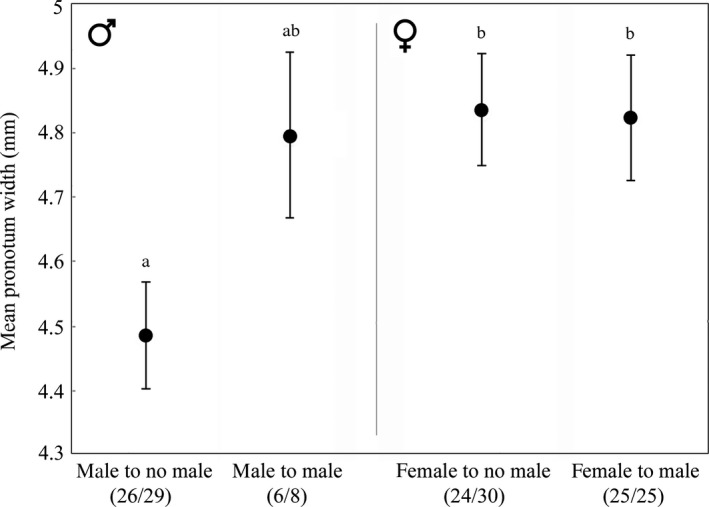
Mean size of beetles arriving at initially unoccupied carcasses placed in the field. Numbers under *x*‐axis groups indicate the number of beetles successfully retrieved for analysis and the total number of beetles recorded in each category: ‘male to no male’: male arriving at carcass without a calling male; ‘male to male’ male arriving at carcass with a previously arrived calling male; ‘female to no male’: female arriving at carcass without a calling male; ‘female to male’ female arriving at carcass with a previously arrived calling male. Lower case letters over means and standard errors indicate Tukey's honest significant differences at *P* < 0.05.

There was no significant deviation from parity in the overall sex ratio of the first beetles to locate carcasses that were unoccupied by calling males (*n *= 29 males; 30 females, *χ* ^2^_1_ **= 0.017, *P *> 0.896). In contrast to experiment 1, only 30% of these carcasses without calling males were discovered in the first 2 days. Males that arrived first at carcasses unoccupied by another male (i.e. they could not have responded to male pheromones) were significantly smaller than female arrivals but did not differ in size from males that arrived at carcasses already occupied by another male (anova, size, *F*_3,77_ **= 3.851, *P *= 0.013; Fig. 3). Our sample of beetles caught in traps revealed no statistically significant difference between the size (pronotal width) of males and females in the wider population [males, 4.78 ± 0.47 mm; females, 4.80 ± 0.44 mm (mean ± SD); two‐samples *t*‐test, *t*_1950_ **= 0.766, *P *= 0.444]. Experimental beetles (in experiment 1) and the population sample in experiment 2 had the same median pronotal width of 4.80 mm.

## Discussion

### Sex ratio treatment and parental investment

We found no evidence in support of our first prediction that males would alter the duration of care they provided in response to prebreeding cues of competition (neither sex ratio bias or the potential density cue in the ‘no cue’ treatment). This is noteworthy in the light of our previous findings that males alter their parental care behaviour (duration of care) in response to variation in the levels of competition experienced when breeding on a carcass (Hopwood *et al*., 2015). Perhaps the most parsimonious explanation for the lack of response seen here is that future reproductive success depends on carcass availability (necessary to translate matings into fitness gains) and therefore fitness benefits or costs associated with responses to actual competition experienced during a breeding event are more likely to be visible to selection. This might explain the finding that our sex ratio treatment affected the pattern of arrival of wild beetles to experimental calling males. Although this is likely to have been mediated by differences in calling behaviour (or pheromone composition), we were unable to discern those differences in this study. In a previous study, we showed that caregiving males respond to the immediate presence of both brood parasitic females and satellite males by extending care and increasing mating frequency (Hopwood *et al*., 2015). Through this reactive behavioural response, males could increase their proportion of both current and future paternity (Müller & Eggert, 1989; House *et al*., 2008). However, prereproductive cues to future reproductive competition may not provide beetles with sufficiently accurate information on which to base future reproductive strategy. This is probably because the level of competition beetles face at each breeding event is unpredictable. Successful beetles are likely to be those that respond effectively to immediate competition and therefore prior sex ratio ‘information’ from the laboratory treatments was effectively ‘discarded’ in favour of more up‐to‐date information. Treatment differences in calling success might correspond with the time taken for males, whose willingness to call may have been inhibited by strong male competition in the laboratory, to reappraise their new situation and adjust their behaviour accordingly.

We predicted that large males would desert broods sooner than small males because their increased prowess in contests for carcasses means they would have more mating opportunities available to them. Instead, we found that smaller males were disproportionately successful among males at attracting females (Fig 1a). The consequence of this was that small males were significantly more likely to breed in a socially monogamous pair (Fig 2a). The mechanism leading to small male success in attracting females preferentially is unclear, but in the related *Nicrophorus orbicollis,* females discriminate between males based on odour (Beeler *et al*., 2002). We also have evidence in *N. vespilloides* that females are more attracted to and prefer to mate with smaller males in the lab (A. J. Moore, unpublished data). Moreover, Walling *et al*. (2009) found that smaller male *N. vespilloides* released pheromones more often than larger males, providing evidence supporting conditional alternative tactics related to body size. Further investigation will be necessary to determine the mechanism underlying the male size‐related pattern of female attraction we report here, but we found no clear evidence that this was influenced by the social environment that males experienced prebreeding.

Laboratory studies of *N. vespilloides* have shown that positive body size differences largely determine dominance status (Bartlett & Ashworth, 1988; Otronen, 1988; Müller *et al*., 1990; Hopwood *et al*., 2013; Lee *et al*., 2013) and therefore reproductive success (Müller *et al*., 2007) on contested carcasses. The conclusions of these laboratory studies were based on the syllogism: (i) big beetles win contests; (ii) contest winners have greater reproductive success; (iii) therefore big beetles have greater reproductive success. However, there is evidence in other taxa that male–male competition and female choice can act in opposition (Simmons, 1991; Moore & Moore, 1999; Petersson *et al*., 1999; Casalini *et al*., 2009). Theoretical work shows that trade‐offs between male parental investment vs. searching for additional mating opportunities are likely to be affected by both frequency of encounters between potential mates and competitors, and intrinsic differences between males in mating success (Kokko & Rankin, 2006; de Jong *et al*., 2012). Our empirical results suggest that small males can achieve success by avoiding direct male–male competitive encounters. This is because when a female arrived first in response to a calling male, the male usually ceased calling, the pair mated, buried the carcass and the likelihood of further arrivals of either sex was reduced. Therefore, although selection may favour large males in the context of male–male competitive encounters for dominant access to carcasses and females, they appear to be less able to attract females, and more likely to attract rival males. This could counteract the competitive advantage held by large males because small males can achieve reproductive success via sneak satellite mating with females at contested carcasses, by mating with females encountered off carcasses and also by gaining exclusive access to the breeding female in monogamous breeding associations more often than do large males.

### Female and male benefits

This nonrandom pattern of breeding associations related to body size of males has important consequences for females as well as males. Calling males that encountered male rivals were larger on average than males that did not attract male competitors (Fig. 2b) while calling males on whose carcasses females faced extra‐pair females were significantly smaller than those with whom females faced no female competitors. These combinations of breeding and competitive associations were determined by the status and sex of the first beetle to discover a carcass. When a male is the first on the scene, he must call for a female. If successful in attracting a female, the pair have a good chance of burying their carcass before further challengers arrive. However, if a calling male attracts another male the outcome is more uncertain. Females may coerce a single male into ceasing calling (Eggert & Sakaluk, 1995), but where there is male–male competition, males may continue calling to attract further females. From the female perspective, it is likely to be highly preferable to avoid contested carcasses. This is because females that encounter rival females incur direct costs either from loss of the carcass or from brood parasitism that reduces their success on the discrete breeding resource (Scott, 1998; Eggert *et al*., 2008; Eggert & Müller, 2011). Moreover, increased mating frequency associated with male–male competition as males strive to protect their paternity has direct costs for female reproductive productivity (Head *et al*., 2014; Hopwood *et al*., 2015). Perhaps the ability of females to effectively prevent smaller males from continuing calling, or small male disinclination to risk calling a male competitor after a female has arrived, was involved in the origin of a female preference based on male size. Regardless of the origin of the preference, the current result seems to show that females attracted to smaller males benefit because they are less likely to face extra‐pair competition.

Although offspring in a breeding pair are sired overwhelmingly by the resident male(s) (e.g. 89% in Müller *et al*., 2007), the proportion of single females breeding alone, using sperm from previous matings, may also be high (e.g. 39% in Eggert, 1992). This is supported by the results of experiment 2 where we found no difference in the frequency of males or females discovering carcasses with no calling male present (29 vs. 30 respectively). These uniparental females represent an opportunity for males to translate off‐carcass matings to fitness gains by calling and mating with females without having found a carcass. Small males in monogamous breeding associations might benefit here as well; our previous research indicated that males in monogamous breeding partnerships deserted offspring earlier than males at contested carcasses (Hopwood *et al*., 2015). These males could potentially desert earlier with little risk to their paternity share in the current brood and, moreover, may stand to gain similar disproportionate success in attracting females even when they call without a carcass.

However, as attractive as they may be, small males do not have it all their own way. Large males are likely to be more resilient to the effects of direct competitors when they do attract a female to a carcass. If larger males are less successful than small males at attracting females when calling without a carcass their best strategy might be to actively seek out competitive encounters at carcasses. Having secured dominance against rivals a male can ensure, by repeated mating, that his paternity is maximized and that females leave with fresh sperm stores (Hopwood *et al*., 2015). Our results suggest a potential additive component to this benefit: a large male attracted to a carcass claimed already by other beetles has a reasonable chance of displacing the resident male. Furthermore, he may then gain not only the carcass but also access to females attracted by the calling of the displaced smaller male. Müller & Eggert (1987) found evidence that calling males did attract other males even in the absence of a carcass but size was not measured. Our results from experiment 2 corroborate this and uncover a trend that might indicate size‐dependent differences in male strategies. Both males and females arrived at unclaimed carcasses but males that were first to arrive were smaller than the population average size (Fig. 3). Male first arrivals were significantly smaller than female arrivals but because fewer males than females were attracted to occupied carcasses, the male comparison is based on a small sample (*n *= 6).

## Conclusion

In nature, the agents of selection are often difficult to identify (Wade & Kalisz, 1990; MacColl, 2011). Studies in burying beetles (e.g. Hopwood *et al*., 2014; Carter *et al*., 2015) and other species (Goldsmith, 1987; Goldsmith & Alcock, 1993) have shown that a competitive advantage associated with body size can be mediated by social context. Our results suggest that when the social environment encountered is itself modified by sexual selection, cues indicating likely future competition are decoupled from individual experience. Smaller male beetles’ disproportionate success in attracting females strongly influences the frequency of intrasexual contests in males and females. Hunt *et al*. (2009) note that it is hard to quantify the combined effects of sexual selection on a targeted trait without detailed knowledge of the direction, strength and interaction of male–male competition and female mate choice. The prediction that large males have a general fitness advantage due to superior competitive ability was not supported in *N. vespilloides* in nature, highlighting the importance of acknowledging alternative routes to fitness that may influence the nature of evolutionary responses.

## Funding

This work was supported by a PhD studentship from the Natural Environment Research Council (NE/1528326/1) and a grant from NERC to N.J.R. and A.J.M. (NE/1025468/1).

## Contribution of authors

PEH collected and analysed the data. All authors designed the experiments and contributed to writing the manuscript.
